# Comparison of ultrasound guidance for axillary or subclavian vein catheterization: a randomized controlled non-inferiority trial

**DOI:** 10.1186/2197-425X-3-S1-A614

**Published:** 2015-10-01

**Authors:** B Louart, G Buzançais, C Roger, L Muller, JY Lefrant

**Affiliations:** Nimes University Hospital, Nimes, France

## Introduction

Axillary vein catheterization appears as an interesting alternative to subclavian vein catheterization (SVC) under ultrasound (US) guidance.[1]

## Objectives

The aim of this trial was to compare the two approaches.

## Methods

Randomized non-inferiority single-centre study. All patients admitted in intensive care unit (ICU) or operating room, requiring a central vein catheterization (CVC) without contraindication for SVC, were randomly assigned to subclavian or axillary groups. The primary endpoint was to compare success rate of each approach. The secondary endpoints were: strategy success rates, catheter position and complications. Strategy of CVC consisted in using the allocated approach and switching to the non-allocated approach after two failed punctures.

## Results

122/132 included patients were analysed (60 and 62 in subclavian and axillary group, respectively). The approach success rates for subclavian and axillary sites were 87.7% and 85.5%, respectively (difference -2.2%, 90%CI [-12.5%-8.1%], non-inferiority p = 0.18). The subclavian and axillary strategy success rates were 96.5% and 98.4%, respectively (difference -1.9%, 90%CI [-4.9%-8.7%], non-inferiority p < 0.01). Thrombogenic catheter positions were 7 (12.3%) in subclavian group vs. 19 (31.7%) in axillary group (p = 0.01). Complications were comparable in the two groups (2 (3.3%) vs. 4 (6.5%), p = 0.68).

## Conclusions

In terms of absolute success rate, axillary is not non-inferior to subclavian approach. In terms of strategy success rate, axillary is non-inferior to subclavian approach. After two failed subclavian approaches, changing for axillary approach leads to 98% success rate. Although associated with more thrombogenic catheter extremity position, axillary approach can be considered as a rescue alternative after subclavian approach failure.

**Table 1:** Success rates for subclavian and axillary groups.

	Subclavian group	Axillary group	p
Success rates (%, 95CI)			
Approach	88.3 [77.4 - 95.2]	85.5 [74.2 - 93.1]	0.202
Strategy	96.7 [88.5 - 99.6]	98.4 [91.3 - 99.7]	0.009
First puncture	66.7 [53.3 - 78.3]	67.7 [54.7 - 79.1]	0.142
